# Assessing the safety, impact and effectiveness of RTS,S/AS01_E_ malaria vaccine following its introduction in three sub-Saharan African countries: methodological approaches and study set-up

**DOI:** 10.1186/s12936-022-04144-3

**Published:** 2022-04-25

**Authors:** Nicolas Praet, Kwaku Poku Asante, Marie-Cecile Bozonnat, Elaine Jacqueline Akité, Patrick Odum Ansah, Laurence Baril, Owusu Boahen, Yolanda Guerra Mendoza, Valerie Haine, Simon Kariuki, Mathieu Lamy, Kenneth Maleta, Randy Mungwira, Latif Ndeketa, Abraham Oduro, Bernhards Ogutu, Fredrick Olewe, Martina Oneko, Mattéa Orsini, Francois Roman, Edith Roset Bahmanyar, Dominique Rosillon, Lode Schuerman, Valentine Sing’oei, Dianne J. Terlouw, Stéphanie Wéry, Walter Otieno, Jean-Yves Pirçon

**Affiliations:** 1 GSK, Wavre, Belgium; 2 Kintampo Health Research Centre, Research and Development Division, Ghana Health Service, Kintampo North Municipality, Kintampo, Ghana; 3 London School of Hygiene and Tropical Medicine, London, UK; 4 Clinics C/O GSK, Wavre, Belgium; 5 Navrongo Health Research Centre, Research and Development Division, Ghana Health Service, Navrongo, Ghana; 6 Institut Pasteur, Phnom Penh, Cambodia; 7 Kenya Medical Research Institute, Centre for Global Health Research, Kisumu, Kenya; 8 Aixial C/O GSK, Wavre, Belgium; 9 University of Malawi College of Medicine, Mangochi, Malawi; 10 Malawi Liverpool Wellcome Trust Clinical Research Programme, Kamuzu University of Health Sciences, Blantyre, Malawi; 11 Centre for Research in Therapeutic Sciences (CREATES), Strathmore University, Nairobi, Kenya; 12 Kenya Medical Research Institute, Centre for Clinical Research, Nairobi, Kenya; 13 HQ Global Clinical, Organon International GmbH, Luzern, Switzerland; 14 KEMRI-Walter Reed Project, US Army Medical Research Directorate-Kenya, Kombewa, Kenya; 15 Liverpool School of Tropical Medicine, Liverpool, UK; 16 Present Address: Janssen Pharmaceutica NV, Beerse, Belgium; 17 Present Address: DESiRE-Consulting, Sorée, Belgium

**Keywords:** Malaria, RTS,S/AS01_E_, *Plasmodium falciparum*, Safety, Effectiveness, Impact, Protocol

## Abstract

**Background:**

Following a 30-year development process, RTS,S/AS01_E_ (GSK, Belgium) is the first malaria vaccine to reach Phase IV assessments. The World Health Organization-commissioned Malaria Vaccine Implementation Programme (MVIP) is coordinating the delivery of RTS,S/AS01_E_ through routine national immunization programmes in areas of 3 countries in sub-Saharan Africa. The first doses were given in the participating MVIP areas in Malawi on 23 April, Ghana on 30 April, and Kenya on 13 September 2019. The countries participating in the MVIP have little or no baseline incidence data on rare diseases, some of which may be associated with immunization, a deficit that could compromise the interpretation of possible adverse events reported following the introduction of a new vaccine in the paediatric population. Further, effects of vaccination on malaria transmission, existing malaria control strategies, and possible vaccine-mediated selective pressure on *Plasmodium falciparum* variants, could also impact long-term malaria control. To address this data gap and as part of its post-approval commitments, GSK has developed a post-approval plan comprising of 4 complementary Phase IV studies that will evaluate safety, effectiveness and impact of RTS,S/AS01_E_ through active participant follow-up in the context of its real-life implementation.

**Methods:**

EPI-MAL-002 (NCT02374450) is a pre-implementation safety surveillance study that is establishing the background incidence rates of protocol-defined adverse events of special interest. EPI-MAL-003 (NCT03855995) is an identically designed post-implementation safety and vaccine impact study. EPI-MAL-005 (NCT02251704) is a cross-sectional pre- and post-implementation study to measure malaria transmission intensity and monitor the use of other malaria control interventions in the study areas, and EPI-MAL-010 (EUPAS42948) will evaluate the *P. falciparum* genetic diversity in the periods before and after vaccine implementation.

**Conclusion:**

GSK’s post-approval plan has been designed to address important knowledge gaps in RTS,S/AS01_E_ vaccine safety, effectiveness and impact. The studies are currently being conducted in the MVIP areas. Their implementation has provided opportunities and posed challenges linked to conducting large studies in regions where healthcare infrastructure is limited. The results from these studies will support ongoing evaluation of RTS,S/AS01_E_’s benefit-risk and inform decision-making for its potential wider implementation across sub-Saharan Africa.

**Graphic abstract:**

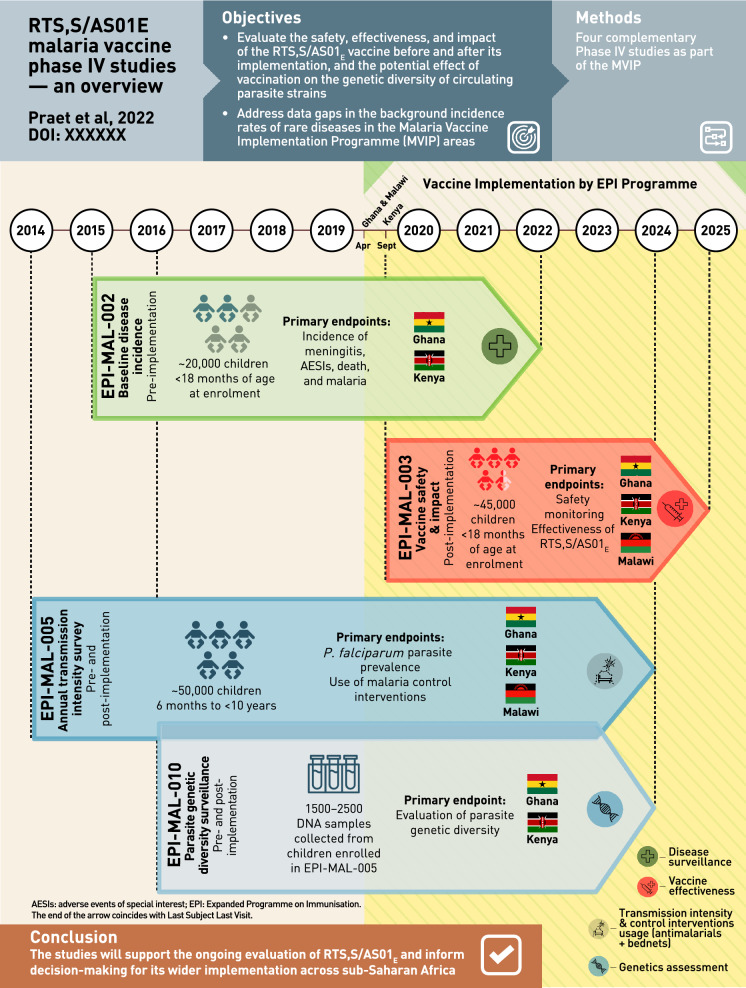

## Background

RTS,S/AS01_E_ (GlaxoSmithKline (GSK), Belgium) is a pre-erythrocytic *Plasmodium falciparum* malaria vaccine developed for routine immunization of young children living in malaria-endemic countries. In the pivotal Phase III trial, 4 doses of RTS,S/AS01_E_ administered to children aged 5 months or older reduced clinical malaria by 39% and severe malaria by 29% over 4 years of follow-up [1, 2]. In addition, vaccination with RTS,S/AS01_E_ was associated with a reduction in overall hospitalizations, and hospitalizations due to malaria, severe anaemia and the need for blood transfusion [1, 3, 4]. RTS,S/AS01_E_ was generally well tolerated and although more reactogenic than control vaccines, local and systemic symptoms were generally transient and mild-to-moderate in intensity [1, 4]. There was a higher incidence of febrile convulsions in RTS,S/AS01_E_ recipients versus controls after vaccination in children aged 5 months or older, with the risk mainly during the first 3 days after vaccination [3]. Three safety signals were identified during the study. In the 5–17 months age group, higher incidences of meningitis (any cause) and cerebral malaria cases were observed in RTS,S/AS01_E_-vaccinated children than in children vaccinated with control vaccines [3]. In addition, there was a gender-specific imbalance in mortality, with higher mortality rates in girls vaccinated with RTS,S/AS01_E_ compared to girls vaccinated with control vaccines, without differences in risk factors, time to death or causes of death that could explain the results. No such difference was observed in boys. Detailed case evaluation indicated that these imbalances were likely to be chance findings due to unexpectedly low rates of meningitis in the control group (1 case in approximately 3000 children followed for almost 4 years), or a low mortality rate in girls in the control group, or the lack of biological plausibility to explain the causal relationship to RTS,S/AS01_E_ vaccination [3].

In 2015, RTS,S/AS01_E_ received a positive scientific opinion from the European Medicines Agency [5]. In a 2016 position paper, the World Health Organization (WHO) acknowledged that several uncertainties related to programmatic feasibility, RTS,S/AS01_E_ impact and safety remained. The WHO, therefore, adopted the recommendations of the Strategic Advisory Group of Experts on Immunization and the Malaria Policy Advisory Committee who jointly endorsed pilot implementation of the vaccine in 3–5 settings in sub-Saharan Africa [6]. Under the programme, 3 vaccine doses are being administered to children 5–9 months of age in areas of moderate-to-high transmission of malaria, with a fourth dose 15–18 months later [6].

In April 2017, the WHO announced that RTS,S/AS01_E_ would be first introduced in selected areas in Ghana, Kenya and Malawi by the respective routine national immunization programmes in the framework of the Malaria Vaccine Implementation Programme (MVIP). Authorization for use of RTS,S/AS01_E_ in this context was granted on 24 April 2018 by the Ghana Food and Drug Board, 11 May 2018 by the Kenya Pharmacy and Poisons Board, and 16 May 2018 by the Malawi Pharmacy and Medicines Regulatory Authority. Vaccination started on 23 April 2019 in Malawi, 30 April 2019 in Ghana and 13 September 2019 in Kenya [7]. RTS,S/AS01_E_ is the first vaccine to be implemented as a complementary tool to existing interventions under the Global Technical Strategy for Malaria, 2016–2030 [8]. In 2022 the WHO recommended that RTS,S/AS01_E_ should be used for the prevention of *P. falciparum* malaria in children living in regions with moderate to high malaria transmission as part of a comprehensive malaria control strategy [9].

The MVIP is a collaboration between selected countries and international private and public partners established by the WHO to coordinate, support and evaluate the introduction of RTS,S/AS01_E._ Key aspects of the MVIP have been summarized by the WHO in an on-line series of question and answers, in a summary of key milestones in the journey to vaccine implementation, and in the 2021 SAGE report of the RTS,S/AS01_E_ vaccine [10–12]. GSK is donating the RTS,S/AS01_E_ vaccine doses necessary to the MVIP (up to 10 million doses) [7]. In addition to RTS,S/AS01_E_ introduction, the MVIP evaluates the vaccine safety, impact, and effectiveness in order to generate information necessary to inform potential future policy for the deployment of RTS,S/AS01_E_ on a broader scale. A first step is the WHO-commissioned Malaria Vaccine Pilot Evaluation (MVPE). This consists of household surveys, and sentinel hospital and community mortality surveillance, building on routine systems. The MVPE will measure the programmatic feasibility of delivering a 4-dose vaccine schedule, vaccine safety in routine use, and the impact of the malaria vaccine on severe malaria and all-cause mortality. This evaluation is largely based on passive follow-up and comparison of the occurrence of vaccine safety and impact study endpoints between vaccine implementation areas (exposed clusters) and areas where the vaccine is not yet implemented (unexposed clusters) [13]. Second, as a part of the MVIP, GSK has designed a comprehensive post-approval plan that includes 4 observational studies to assess RTS,S/AS01_E_ vaccine safety, effectiveness, impact, and the potential effect of vaccination on the genetic diversity of circulating parasite strains. This paper presents an overview of GSK’s post-approval plan currently being conducted in the MVIP areas [11]. The challenges associated with conducting large observational studies in regions with limited healthcare infrastructures are discussed, as well as the opportunities to leverage existing collaborations, research infrastructure and global expertise. Over the coming years, these studies will contribute to the ongoing assessment of the RTS,S/AS01_E_ benefit-risk profile, and to informing decisions for its potential wider implementation across malaria endemic areas of Africa.

### Overview of GSK’s RTS,S/AS01_E_ post-approval plan

**Fig. 1:**
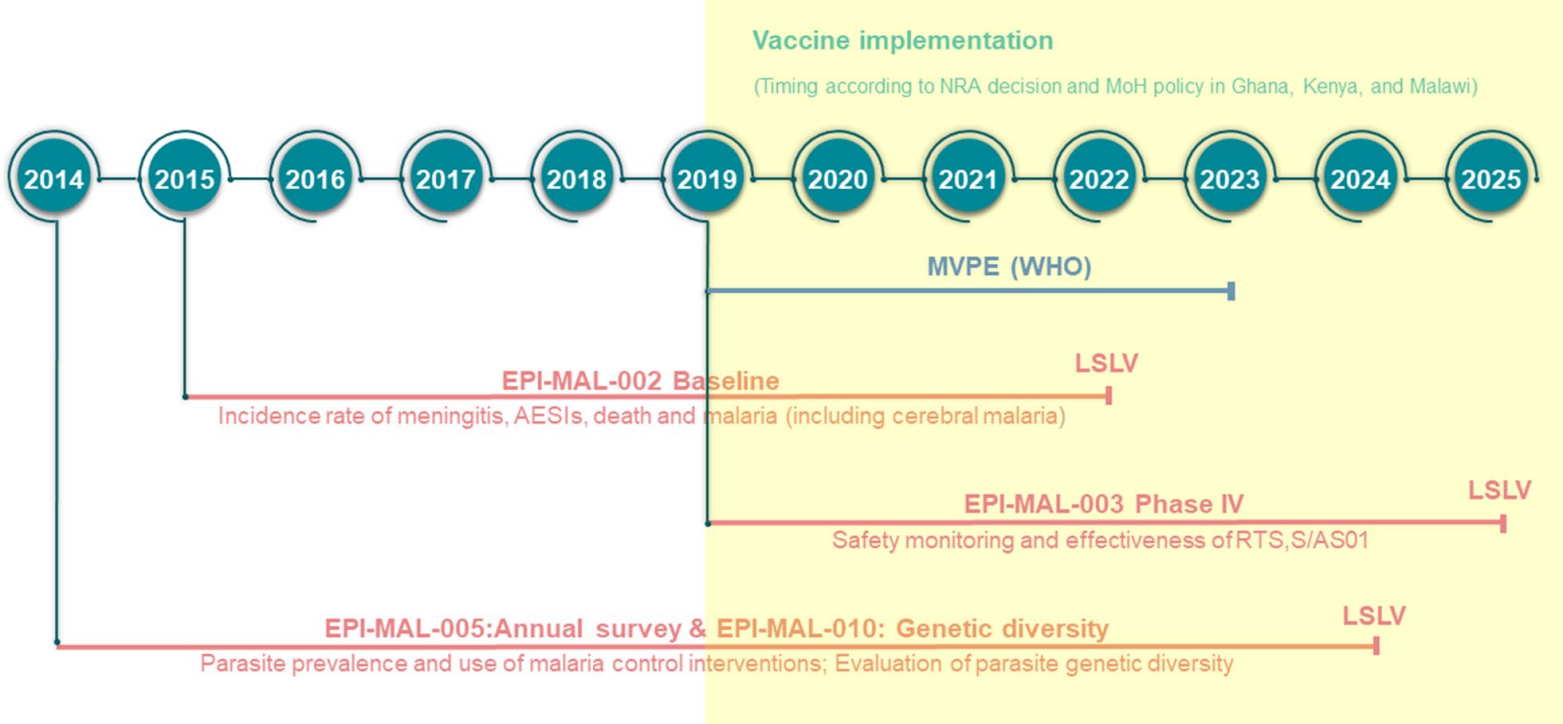
Overview GSK’s RTS,S/AS01_E_ vaccine post-approval plan embedded within the Malaria Vaccine Implementation Programme. *AESIs* adverse events of special interest, *LSLV* last subject last visit, *MoH* Ministry of Health, *MVPE* Malaria Vaccine Programme Evaluation, *WHO* World Health Organization, *NRA* National Regulatory Authority

Many low and lower-middle income countries where malaria is endemic have little or no data on background incidence rates of rare diseases such as those that may be reported as adverse events following immunization. This may prevent robust post-authorization vaccine safety and effectiveness monitoring, and can lead to delays in detecting safety signals, potentially contributing to vaccine hesitancy related to the new vaccine, or to other vaccines introduced in the future. In these settings, disease surveillance studies conducted prior to vaccine introduction can be used to determine reliable background rates of specific diseases/events that can be compared with post-introduction observations. In order to further monitor the benefit-risk profile of RTS,S/AS01_E_, GSK’s post-approval plan is designed to assess vaccine safety, impact, and effectiveness in a real-life setting. It comprises 4 GSK-sponsored Phase IV studies (Fig. 1) including a before-after comparison in which data collected in the pre-RTS,S/AS01_E_ vaccine introduction study, EPI-MAL-002 (NCT02374450), and the post-vaccine introduction study, EPI-MAL-003 (NCT03855995), are compared. For operational reasons, the before-after comparison is being conducted in Ghana and Kenya only. In addition, because the Ministries of Health of the implementing countries are not introducing RTS,S/AS01_E_ into all national areas, vaccine safety and impact will also be assessed using a contemporaneous comparison between exposed and unexposed areas of the study endpoints. Moreover, since annual and/or geographical variations in malaria incidence may occur as a result of changes in malaria transmission intensity or in malaria control intervention coverage, these potential confounders are monitored in the EPI-MAL-005 study (NCT02251704).

EPI-MAL-005 is an observational cross-sectional study assessing *P. falciparum* parasite prevalence (as a proxy for transmission intensity) and malaria control intervention coverage over 10 annual surveys in the EPI-MAL-002 and EPI-MAL-003 study areas. Results from EPI-MAL-005 will be used to adjust the temporal and contemporaneous comparison analyses for potential year-to-year variation during the conduct of EPI-MAL-002 and EPI-MAL-003. Finally, because *P. falciparum* has evolved multiple mechanisms to vary cell surface antigens and evade the host’s immune response, an ancillary study to EPI-MAL-005, EPI-MAL-010 (EUPAS42948), has been designed to monitor parasite genetic diversity before and after vaccine implementation.

### Assessment of vaccine safety, effectiveness and impact (EPI-MAL-002 and EPI-MAL-003)

**Fig. 2:**
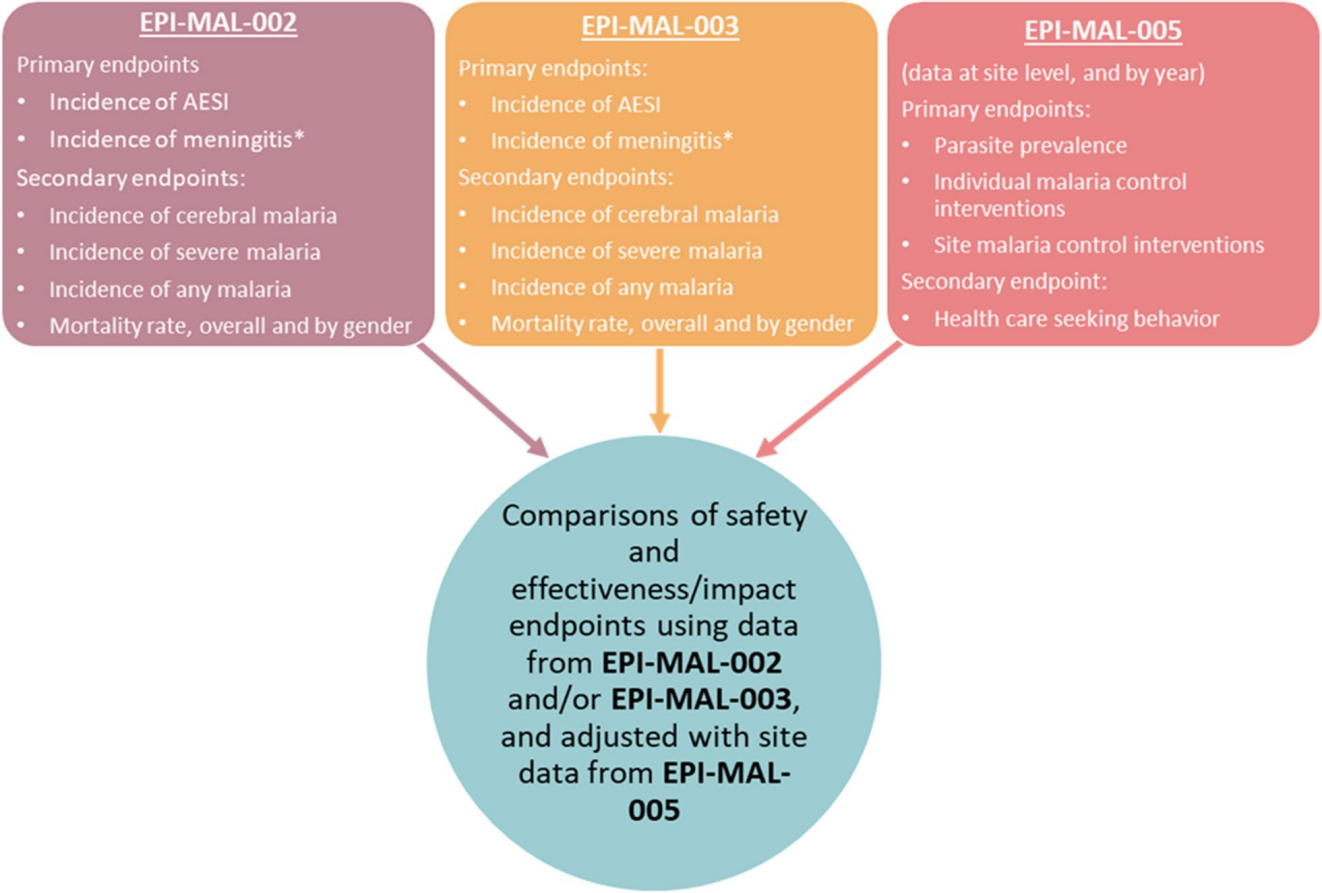
Evaluation of safety, effectiveness and impact using data collected as part of the RTS,S/AS01_E_ post-approval plan. *AESI* adverse events of special interest. Asterisk indicates the potential risk of meningitis will be monitored on ongoing basis using the maximized sequential probability ratio test (MaxSPRT) method

Study EPI-MAL-002 is designed to collect incidence data of pre-defined health outcomes, i.e., adverse events of special interest (AESI). These include rare events potentially associated with vaccination (Table 1), meningitis, malaria (including severe malaria and cerebral malaria), death and other health outcomes leading to hospitalization, before RTS,S/AS01_E_ vaccine introduction. To assess vaccine safety, effectiveness and impact, these baseline incidence rates will be compared with rates documented in the post-implementation study EPI-MAL-003 which commenced when RTS,S/AS01_E_ vaccination was introduced by the Ministries of Health (Fig. 2). RTS,S/AS01_E_ vaccine implementation follows a phased introduction in which RTS,S/AS01_E_ is introduced into some areas (exposed clusters) but not others (unexposed clusters). Thus, in addition to the before-after comparison, EPI-MAL-003 also includes a contemporaneous comparison of endpoints of interest between vaccinated and unvaccinated study participants.

**Table 1:** Summary of safety endpoints for evaluation in studies EPI-MAL-002 and EPI-MAL-003

Study endpoints	Event
Adverse events of special interest*	Acute disseminated encephalomyelitis, encephalitis, Guillain-Barré syndrome, generalized convulsive seizure, hypotonic hypo-responsive episode, intussusception, hepatic insufficiency, renal insufficiency, juvenile chronic arthritis, Stevens Johnson syndrome and toxic epidermal necrolysis, Henoch Schonlein purpura, Kawasaki disease, diabetes mellitus type 1, thrombocytopenia and anaphylaxis
Meningitis*	Etiology confirmed meningitis, etiology confirmed, probable and clinically suspected meningitis, clinically suspected meningitis
Malaria	Any malaria, severe malaria, cerebral malaria
Deaths	Deaths all causes, death all causes: female/male
Other adverse events leading to hospitalization	Anemia, gastroenteritis, lower respiratory tract infection, sepsis, upper respiratory tract infection, skin infection, malnutrition, conjunctivitis, helminthic infection, urinary tract infection, bacterial infection, burn

*Co-primary endpoints

#### Study design and population of EPI-MAL-002 and EPI-MAL-003

GSK’s Phase IV study package is fully embedded in the MVIP. Therefore, selection of participating clusters depended on the cluster identification process led by the Ministries of Health according to the WHO guidance [14]. EPI-MAL-002 is being conducted in Ghana and Kenya and EPI-MAL-003 in Ghana, Kenya and Malawi. There are 4 study sites (corresponding to 4 clusters of the MVIP) in each country, of which 2 became exposed clusters and 2 became unexposed clusters in EPI-MAL-003. The EPI-MAL-002 and EPI-MAL-003 studies will enroll prospective cohorts of approximately 20,000 and 45,000 children, respectively. The areas participating in EPI-MAL-002 became exposed clusters in EPI-MAL-003 once vaccination commenced, to allow the before-after comparison. EPI-MAL-003 added additional unexposed clusters to allow the contemporaneous comparison. Additionally, both exposed and unexposed clusters were added in Malawi that was not included in study EPI-MAL-002.

To allow direct comparisons of endpoints, EPI-MAL-002 and EPI-MAL-003 are strictly identical in terms of design and conduct. Both are multi-country observational studies with prospective cohort event monitoring. Children < 18 months of age are enrolled during routine immunization with the pentavalent diphtheria-tetanus-pertussis-hepatitis B and *Haemophilus influenzae* type b vaccine, through direct invitation or when hospitalized before routine immunization. Similarly, EPI-MAL-003 is enrolling children presenting for routine immunization, regardless of their future vaccination status with RTS,S/AS01_E_.

In both studies, follow-up until approximately 5 years of age (corresponding to around 2 years after the fourth dose of RTS,S/AS01_E_ in children in the exposed clusters in EPI-MAL-003) consists of active surveillance for outpatient and inpatient visits by each enrolled participant and of 10 home visits conducted according to a specific time frame (prospective cohort). In addition, hospital-based disease surveillance is organized across the entire study area for infants and children who are not enrolled in the prospective cohort. In other words, throughout the whole study period, all hospitalized children under the age of 5 years who are not already enrolled in the prospective cohort and who live within the study areas are eligible for enrolment in the hospital-based disease surveillance.

#### Objectives and endpoints

The study objectives are to estimate the incidence of protocol-defined events, including AESI, meningitis, other adverse events leading to hospitalization, death, and malaria (including severe malaria and cerebral malaria). AESI (Table 1) are events that have historically been associated with other vaccines, that may be hypothetically associated with RTS,S/AS01_E_ given that this vaccine has relatively new components compared to other widely used vaccines, or that were identified from the results of the Phase III efficacy study (meningitis, severe malaria and cerebral malaria, gender-specific mortality) [1, 3]. Estimated incidences will be used to monitor (1) vaccine safety and (2) vaccine effects (direct, indirect, total and overall effects) on the incidence of any malaria, severe malaria, cerebral malaria, all-cause hospitalizations, malaria-attributable hospitalizations, the prevalence of anaemia in hospitalized children, and the mortality rate. The direct effect (effectiveness) of RTS,S/AS01_E_ will compare malaria-related events in vaccinated and unvaccinated children from exposed clusters enrolled in active surveillance. Vaccine impact (indirect, total and overall effects) will be investigated by comparing the incidence of malaria-related events in unvaccinated children (either from EPI-MAL-002 or from EPI- MAL-003 unexposed clusters) with the incidence of events in children from EPI-MAL-003 exposed clusters (either unvaccinated children [indirect effect], vaccinated children [total effect] or both [overall effect]).

#### Methods and analysis

The studies are conducted in the setting of routine medical practice and local laboratory testing (first-line testing), and include a strong support component comprised of study-specific trainings, telemedicine support, second-line laboratory testing, and consultation with an external expert panel for final case classification. These tools are likely to enhance case detection and ascertainment rates. Both EPI-MAL-002 and EPI-MAL-003 use identical surveillance in order to allow a robust comparison of study outcomes before and after the RTS,S/AS01_E_ vaccine introduction.

Freely given and written or witnessed and thumb-printed informed consent is obtained from each study participant’s parent/legal representative prior to study participation. Protocol-specified procedures include the collection of demographic data, vaccination records, medical history, medical care episodes, adverse events, use of malaria control measures, development delays or death, and a physical examination when contacts between study staff and subjects occur. The protocol requires collection of a sample of 5 ml of blood for testing at an external laboratory (second-line laboratory) in all suspected cases of AESI and meningitis. All study participants are treated according to good medical and routine practices [15]. In cases where a lumbar puncture is performed according to routine medical practice, when possible an aliquot of cerebro-spinal fluid (CSF) is required to be sent to the second-line laboratory for testing. An independent panel of external medical experts perform blinded reviews of all suspected cases of meningitis and cerebral malaria, as well as any case of any other endpoint for which the diagnosis is equivocal. The cause of death is determined from medical records when available, or through verbal autopsy for children who die in the community.

Incidence rates of safety endpoints will be calculated and compared based on both before-after and between cluster comparisons using a univariable and multivariable Poisson regression model adjusted for specific covariates, including if applicable, those identified in EPI-MAL-005. The vaccination status of participants will be confirmed using data from individual vaccination cards, vaccination registers and health and demographic surveillance system (HDSS) or equivalent surveillance system. Incidence rates will be computed using person-time denominators for group of interest. Each child will contribute person-time until study end or 5 years of age, whichever occurs first.

In addition, the potential risk of meningitis is being monitored in near real time using the maximized sequential probability ratio test (MaxSPRT) method [16]. MaxSPRT is a continuous sequential test that allows an early detection of safety event signals. Among vaccinated subjects, the maximum likelihood and the log-likelihood ratios are estimated each month if new meningitis cases are detected. The upper limit is estimated based on the results (meningitis incidence estimate) of EPI-MAL-002. If the log-likelihood ratio reaches a critical value, the comparison between vaccinated and unvaccinated study participants will be done. If the signal is confirmed, further investigation will be performed with additional focus on the subset of children diagnosed with meningitis.

Vaccine effectiveness (direct effect) and impact (overall, indirect, and total effects in the same population) of RTS,S/AS01_E_ on the incidence of malaria will be estimated [17]. Cases of any malaria identified during outpatient visits and hospitalizations at all healthcare facilities will be expressed per person-year of observation for contributing enrolled children.

EPI-MAL-002 started in January 2016 and an interim analysis has been conducted to provide preliminary results from 14,329 children who participated in the prospective cohort monitoring prior to the introduction of RTS,S/AS01_E_ vaccination [18]. EPI-MAL-003 started in March 2019 and is currently ongoing.

### Estimating malaria transmission intensity and the use of malaria control interventions (EPI-MAL-005)

It is expected that the use of RTS,S/AS01_E_ will lead to a reduction in the incidence of malaria disease in vaccinated subjects in EPI-MAL-003 compared to baseline rates recorded in EPI-MAL-002. However, annual fluctuations in malaria incidence occur due to changes in transmission intensity influenced by rainfall patterns or changes in how malaria control interventions are used. By monitoring malaria transmission intensity and the coverage of malaria control interventions in the EPI-MAL-002 and EPI-MAL-003 study sites during the conduct of these studies, EPI-MAL-005 will allow a more accurate estimate of the true impact of RTS,S/AS01_E_ vaccination. This study will contribute to the analysis of vaccine effectiveness and vaccine impact by identifying variables to be used as covariates to adjust the before-after and between cluster comparisons.

#### Study design and population

EPI-MAL-005 has a cross-sectional design with yearly surveys coinciding with the recruitment and follow-up periods of the EPI-MAL-002 and EPI-MAL-003 studies. At each survey, 600 participants aged 6 months to < 10 years are randomly selected from the sites participating in the survey in that year, stratified by age group. The selection process will be repeated every year meaning that the subjects will be different in each cross-sectional survey except if they are re-selected in a subsequent survey by chance. The surveys occur during the period of peak malaria transmission that varies from end September to mid-December in western African sites, from end April to mid-August in eastern and southern African sites [19, 20].

#### Objectives and endpoints

The co-primary study objectives are estimation of *P. falciparum* parasite prevalence (in order to characterize malaria transmission intensity), and of the use of malaria control interventions (insecticide-treated nets, long-lasting insecticidal nets, indoor residual spraying, seasonal malaria chemoprevention, intermittent preventative treatment in infants, and artemisinin-based combination therapy).

#### Methods and analysis

At each survey, demographic information, medical and vaccination history, information on healthcare-seeking behaviors in the previous 14 days, fever in the last 24 h, and use of malaria control measures (bednets for the night before the visit, coils/repellents over the previous 7 days, anti-malaria medication over the previous 14 days), are recorded for all participants. During the survey visit, axillary body temperature is measured, and a capillary blood sample is collected for malaria testing by microscopy and nucleic acid amplification tests (both asexual and sexual parasitaemia). In the event of fever at the time of the visit, or fever or other symptoms/signs of clinical malaria reported in the previous 24 h, a rapid diagnostic test is conducted, and the participant treated if the test is positive. Participants identified as being parasite-positive following microscopy are treated according to national guidelines.

Parasite prevalence and use of malaria control measures are computed by study site, age group, RTS,S/AS01_E_ vaccination status and gender. Annual fluctuations in parasite prevalence are estimated using the Cochran-Armitage trend test. The agreement between parasitaemia as measured by microscopy versus nucleic acid amplification tests is assessed using the Cohen’s Kappa coefficient and the Landis and Koch scale. A risk factor analysis for malaria infection is conducted using a multivariable logistic regression analysis.

The study started in October 2014 and the first two surveys have been published [21]. At the time of the study completion, data from approximately 50,000 participants will be available, providing a comprehensive picture on malaria prevalence variations across the study sites.

### Monitoring *P. falciparum* genetic diversity (EPI-MAL-010)

Although the central NANP amino acid repeat sequence of the circumsporozoite protein used as a major component of the RTS,S vaccine antigen is normally well conserved across parasite strains, *P. falciparum* is a pathogen with high variability and a high number of different circulating haplotypes. The parasite uses numerous mechanisms to vary cell surface antigens and evade the host immune response. For this reason, there is a potential concern that widespread implementation of RTS,S/AS01_E_ could drive the selection of specific parasite variants or alter the number of parasite haplotypes over time by exerting selective pressure. EPI-MAL-010 is an ancillary study to EPI-MAL-005 that will monitor the genetic diversity of circumsporozoite protein sequences in the *P. falciparum* parasite population before and after vaccine implementation.

#### Study design and population

EPI-MAL-010 has a longitudinal, cross-sectional study design and uses capillary blood samples collected from participants enrolled in EPI-MAL-005 over 7 survey years. Samples are from participants aged 6 months to < 5 years with *P. falciparum* parasitaemia confirmed by microscopy and/or nucleic acid amplification tests, and collected before and after RTS,S/AS01_E_ implementation in two study sites: Kintampo (Ghana) in Western Africa and Kombewa (Kenya) in Eastern Africa.

#### Objectives and endpoints

This study is estimating *P. falciparum* haplotype prevalence (i.e., the proportion of participants infected with a specific haplotype) and frequency (i.e., the proportion of a specific haplotype among all detected malaria clones) in participants aged 6 months to < 5 years vaccinated or not with RTS,S/AS01_E_.

#### Methods and analysis

Amplicon sequencing is conducted on samples tested positive for *P. falciparum* by microscopy and/or nucleic acid amplification tests. Trends in the prevalence of specific *P. falciparum* haplotypes with a frequency of at least 5% will be assessed by a logistic regression model. Multinomial logistic regression will be used to describe the annual fluctuations in haplotype frequency using the 3D7 haplotype as the reference group.

### Study set-up opportunities and challenges

Considering the specificities of the study setting, GSK together with its local scientific partners, conducted comprehensive study feasibility assessments in which both scientific and operational aspects were carefully balanced to allow generation of robust data. During the planning phase, global experts provided advice on study design and execution. Evolving circumstances, external constraints and the involvement of multiple stakeholders required adaptability and flexibility, balancing an optimal study design with real-world constraints. Designing, setting-up and conducting a complex post-approval plan in sub-Saharan Africa brings some important setting-specific considerations: (1) data collection needs to be performed using prospective follow-up because existing data collection systems and databases may be sub-optimal or absent; (2) vaccine safety, effectiveness and impact data have to be collected in a healthcare environment where case detection and ascertainment may be challenging because of limited healthcare infrastructure and diagnostic capability; (3) there may be limited access to health care in remote settings; (4) background incidence rates of many diseases including study-specific endpoints are limited or not available; (5) standard laboratory procedures may not exist across all study sites; and (6) sample storage and transportation from remote locations can be challenging.

More information on key parameters that were considered in the framework of the study feasibility assessment is provided below. Study site selection was based on specific criteria: (1) the existence of pre-existing scientific research infra-structure capable of expanding beyond routine data collection. Several of the finally selected study sites have research experience in conducting clinical trials with RTS,S/AS01_E_, and have developed capability in terms of training, experience and quality of healthcare that resulted from previous study participation. However, for some sites without such past experience, additional investment was required to set up baseline structures and procedures; (2) the existence of a HDSS, part of the International Network for the Demographic Evaluation of Populations and Their Health (INDEPTH), or of an equivalent surveillance system. HDSS sites have a demographic database in place that updates, on a regular basis, the number of births, deaths, immigrations and emigrations, and potentially vaccinations and population health outcomes. The number of HDSS sites in Ghana, Kenya and Malawi being limited, a population census had to be fully established or partially enhanced in approximately half of the study sites.

The EPI-MAL-002 and EPI-MAL-003 studies are mainly based on data collection in the framework of routine medical practice, which may hamper the ascertainment of diseases requiring more advanced diagnostic tools. A full understanding of the structure and capacity of the healthcare system in each country where the studies were planned was required, including the assessment of the capacities for case detection and the ascertainment of study endpoints in each study site. As an outcome of this assessment, specific tools were put in place during the study preparation and conduct to enhance case detection and diagnostic capabilities. These include regular and ongoing medical and pharmacovigilance trainings, telemedicine support (Réseau en Afrique Francophone pour la Télémédecine, Switzerland; Agence de Médecine Préventive, Ivory Coast), the distribution of job aids, the support of local laboratories and the set-up of a central reference laboratory (Clinical Laboratory Services, South Africa) for blood and CSF testing. These enhancement tools should increase the likelihood of reaching the highest possible level of diagnostic certainty. For the key endpoint of meningitis, medical and non-medical staffs have received training on meningitis case detection and ascertainment, which includes information on the national guidelines for case management (including lumbar puncture and testing of CSF) with secondary testing to be performed at a central laboratory when sample volume permits.

This multi-country initiative involves key local and global public health partners. The scientific and operational constraints and the complexity of the study set-up provide opportunities for collaborations and alignment in healthcare approaches between countries to promote best practice. However, turnover of trained personal in the study areas is likely to be an ongoing challenge.

Within countries, the MVIP, and more specifically the study design, set-up and conduct, promote collaboration between different authorities, such as routine healthcare system, diagnostic services, national immunization services, epidemiological research, and National Malaria Control Programmes.

Despite best laid plans, the coronavirus disease 2019 (COVID-19) pandemic in Africa continues to unfold and its impact is evolving. In this environment, the MVIP is continuing and measures are being taken to protect the welfare and safety of participants and study staff, and to ensure data integrity [22]. No major change in the rate of RTS,S/AS01_E_ vaccination has been observed during the pandemic thus far, although it might be expected that hospitalization practices may change in order to limit admissions to children with serious or critical conditions and avoid hospital crowding. However, the potential impact of COVID-19 on the evaluation of AESI as determined in pre-vaccination pre-COVID-19 period requires continuous monitoring of the situation. Sensitivity analyses may be conducted considering COVID-19 pandemic periods for both studies.

## Conclusions and perspectives

The implementation and the safety, effectiveness and impact evaluation of RTS,S/AS01_E_ in a real-life setting is a unique and complex undertaking that requires the establishment of large-scale and strong partnerships. Assessing the benefit-risk profile of a 3-dose primary schedule vaccine with a 4^th^ dose booster administered beyond the usual Expanded Programme on Immunization schedule in low and lower-middle income countries requires the establishment of ad-hoc tools and quality control methods to allow collection of robust and reliable data. Whilst technical and operational expertise is key to achieve this goal, human and financial resource needs should not be underestimated. The effort has been based on the WHO-led MVIP, existing research platforms and expertise in the implementing countries, and significant commitment by GSK. The experience with RTS,S/AS01_E_ in sub-Saharan Africa highlights important aspects to be considered when planning and implementing a vaccine post-approval plan in low and lower-middle income countries. The RTS,S/AS01_E_ experience will pave the way for the development and implementation of new generation malaria vaccines, and of other vaccines for the developing world.

## Data Availability

GSK makes available anonymized individual participant data and associated documents from interventional clinical studies which evaluate medicines, upon approval of proposals submitted to www.clinicalstudydatarequest.com. To access data for other types of GSK sponsored research, for study documents without patient-level data and for clinical studies not listed, please submit an enquiry via the website.

## Abbreviations

AESI: Adverse events of special interest
COVID-19: Coronavirus disease 2019
CSF: Cerebrospinal fluid
GSK: GlaxoSmithKline Biologicals SA
HDSS: Health and demographic surveillance system
INDEPTH: International Network for the Demographic Evaluation of Populations and Their Health
MaxSPRT: Maximized sequential probability ratio test
WHO: World Health Organization
MVPE: Malaria Vaccine Programme Evaluation
MVIP: Malaria Vaccine Implementation Programme
